# Pharmacological ascorbate as a novel therapeutic strategy to enhance cancer immunotherapy

**DOI:** 10.3389/fimmu.2022.989000

**Published:** 2022-08-22

**Authors:** Amira Zaher, Laura M. Stephens, Ann M. Miller, Stacey M. Hartwig, Jeffrey M. Stolwijk, Michael S. Petronek, Zeb R. Zacharias, Thaddeus J. Wadas, Varun Monga, Joseph J. Cullen, Muhammad Furqan, Jon C. D. Houtman, Steven M. Varga, Douglas R. Spitz, Bryan G. Allen

**Affiliations:** ^1^ Cancer Biology Program, Holden Comprehensive Cancer Center, The University of Iowa, Iowa City, IA, United States; ^2^ Department of Microbiology and Immunology, The University of Iowa, Iowa City, IA, United States; ^3^ Free Radical and Radiation Biology Program, Department of Radiation Oncology, Holden Comprehensive Cancer Center, The University of Iowa, Iowa City, IA, United States; ^4^ Human Immunology Core & Holden Comprehensive Cancer Center, The University of Iowa, Iowa City, IA, United States; ^5^ Department of Radiology, Holden Comprehensive Cancer Center, The University of Iowa, Iowa City, IA, United States; ^6^ Division of Hematology, Oncology, and Blood & Marrow Transplantation, Department of Internal Medicine, Holden Comprehensive Cancer Center, The University of Iowa, Iowa City, IA, United States; ^7^ Department of Surgery, Holden Comprehensive Cancer Center, The University of Iowa, Iowa City, IA, United States

**Keywords:** pharmacological ascorbate, immune regulation, anti-PD-1, cancer immunotherapy, immune checkpoint inhibitors, antioxidant therapy, prooxidant therapy

## Abstract

Pharmacological ascorbate (i.e., intravenous infusions of vitamin C reaching ~ 20 mM in plasma) is under active investigation as an adjuvant to standard of care anti-cancer treatments due to its dual redox roles as an antioxidant in normal tissues and as a prooxidant in malignant tissues. Immune checkpoint inhibitors (ICIs) are highly promising therapies for many cancer patients but face several challenges including low response rates, primary or acquired resistance, and toxicity. Ascorbate modulates both innate and adaptive immune functions and plays a key role in maintaining the balance between pro and anti-inflammatory states. Furthermore, the success of pharmacological ascorbate as a radiosensitizer and a chemosensitizer in pre-clinical studies and early phase clinical trials suggests that it may also enhance the efficacy and expand the benefits of ICIs.

## 1 Introduction

Immune evasion is one of the cancer hallmarks, defined as a mechanism by which cancer cells either suppress the immune response or mask themselves from immune detection and destruction (1, 2). Anti-tumor immunity is crucial during cancer initiation, progression, and metastasis. Tumors develop multiple mechanisms to evade anti-tumor immunity depending on the level of immune infiltration in the tumor microenvironment that defines it canonically as “cold” or “hot” (3). Cold tumors are considered “immune deserts” characterized by low immune cell infiltration (3). The decreased immunogenicity of cold tumors enables them to evade immune destruction by decreasing levels of tumor antigens, inhibiting maturation of dendritic cells, and suppressing T cell activation, infiltration, and migration (3). Hot tumors are highly infiltrated by immune cells; however, they evade anti-tumor immunity through impaired T cell tumor recognition, increased immune suppressor cell activation, and increased expression of inhibitory immune checkpoint molecules such as programmed death-ligand 1 (PD-L1) (3).

The work of James P. Allison and Tasuku Honjo in the 1990s and early 2000s led to the discovery of immune checkpoint inhibitors (ICIs). ICIs are drugs or antibodies that bind immune checkpoint proteins and prevent the activation of their immune-suppressive function, subsequently leading to increased immune activity. In 2011, Ipilimumab (anti-cytotoxic T-lymphocyte antigen 4 or anti CTLA-4) was the first ICI to receive FDA approval after demonstrating positive clinical outcomes in melanoma patients (4). In 2014, the programmed death protein 1 (anti PD-1) Nivolumab and Pembrolizumab received FDA approval following successful clinical trials in metastatic melanoma (5).

ICIs have since expanded from melanoma to multiple malignancies such as lung, bladder, breast, kidney, and head and neck cancers, etc. (6). However, their application in clinical settings faces numerous challenges such as primary and acquired resistance, toxicity, and limited response in some patient populations. Therefore, it is essential to develop new approaches that increase the sensitivity of tumors to ICIs to meet the clinical need. One approach to overcome these ICI limitations is pharmacological ascorbate (intravenous infusions of vitamin C resulting in ~ 20 mM concentrations in plasma (7)).

Ascorbate (L-ascorbic acid, C_6_H_8_O_6_^-^) is a six-carbon small molecule antioxidant with two ionizable hydroxyl groups and a molecular mass of 176.12 grams discovered by Szent-Györgyi and colleagues in the 1930s (8, 9). Commonly known as vitamin C, ascorbate is an essential micronutrient for humans who need to meet a daily intake of 100 – 200 mg *via* consuming fruits and vegetables (10). Humans are not capable of ascorbate biosynthesis due to a mutation that prevents the conversion of L-gulonolactone to L-ascorbic acid (11, 12). Without adequate ascorbate intake, the homeostasis of multiple physiological functions is disturbed. Ascorbate is a donor antioxidant capable of undergoing two one-electron oxidations; this function plays a central role in collagen biosynthesis and elasticity, redox regulation, enzyme function (as a cofactor), hormone regulation and biosynthesis, and amino acid metabolism (13). As a cofactor, ascorbate functions as an epigenetic regulator for enzymes such as Ten-eleven translocation (TET) proteins and Jumonji C domain-containing histone demethylases (JHDMs) (14–16). By reducing Fe^3+^ to Fe^2+^ ascorbate promotes DNA demethylation, thus epigenetically modifying gene expression in multiple cell populations including cancer cells (as an anticancer effector) and immune cells (14–22).

At a physiological state with adequate dietary vitamin C intake, ascorbate concentrations in the plasma are ≤ 0.08 mM (23). With intravenous administration, ascorbate has unique pharmacokinetics that affects its final plasma concentration and urinary excretion. Oral ascorbate administration with doses up to 6 g/day yields plasma concentrations ≤ 0.22 mM due to the presence of tight physiologic control that leads to urinary excretion of excess ascorbate (23–25). Interestingly, administering ascorbate intravenously can yield plasma concentrations of 15-20 mM temporarily bypassing the strict ascorbate transporter regulation plasma concentrations (7, 23, 25). A remarkable outcome of these unique ascorbate pharmacokinetics is the selective cancer cell killing millimolar plasma concentrations, whilst sparing non-malignant cells, making it an emerging potential anticancer therapy with a plethora of clinical applications (23, 25, 26).

This review discusses the potential of pharmacological ascorbate as a sensitizer for cancer immunotherapy. We discuss the role of ascorbate in the context of immune function and immune regulation, review the most recent findings combining pharmacological ascorbate with ICIs, and highlight potential future directions for this promising approach.

## 2 Ascorbate and immune regulation

Because of ascorbate’s potential to enhance ICIs in cancer therapy, it is imperative to evaluate the effects of ascorbate on the immune function. However, experimental evidence regarding the effects of pharmacological dosing of ascorbate on immune function remains limited but a great deal of information can be learned from previous studies of the effects of ascorbic acid on cells of innate and the adaptive immune systems and how it orchestrates immune function by acting on their proliferation, differentiation, and cytokine secretion.

### 2.1 Ascorbate and the innate immune system

#### 2.1.1 Macrophages and monocytes

Macrophages are phagocytic tissue-differentiated monocytes essential for innate immune defense (27). The main functions of macrophages include phagocytosis, cytokine secretion, and professional antigen presentation to the adaptive immune system (28). In the tumor microenvironment, the role of macrophages can be profoundly influenced by their polarization status which determines their effector functions. Based on their polarization, tumor-associated macrophages (TAMs) can be broadly categorized into M1 and M2 macrophages. M1 macrophages are pro-inflammatory and exert anti-tumor effects by secreting IL-1β, TNF, IL-12, and IL-18 while the several subtypes of M2 macrophages are anti-inflammatory and promote tumor progression by secreting IL-4, IL-10, IL-13, and TGF-β (28–30). Monocytes and macrophages accumulate high ascorbate concentrations (4-6 mM) by upregulating the ascorbate transporter, SVCT2 (31–35). In these cells, ascorbate is a cofactor for the hydroxylase enzyme responsible for regulating the hypoxia-inducible factors (HIF) family (22) whose activation impacts the polarization of TAMs towards the M2 phenotype that suppresses anti-tumor immunity and the associated T cell cytotoxicity (36–39). Additionally, some evidence in macrophages pointed at TET proteins and Jumonji C domain-containing histone demethylases that use ascorbate (21, 22) suggesting that ascorbate may play a role in macrophage function and polarization through epigenetic regulation.

Generally, ascorbate plays a pro-phagocytic role that promotes monocyte survival, protects against reactive oxygen species (ROS), and enhances phagocytosis (32, 40). A study by Oberritter and colleagues showed that ascorbate levels in macrophages decrease from baseline after phagocytosis, indicating that it is required to protect macrophages from ROS generated during phagocytosis (41). Monocytes treated with ascorbate (4-20 mM) were protected from FAS-induced cell death, thus promoting survival and differentiation (42). Ascorbate can also exert an anti-inflammatory effect on macrophages. In an *in vitro* study, ascorbate reduced the secretion of the pro-inflammatory cytokines, TNF, and IL-6 (43). Thus, ascorbate may selectively drive macrophage activity in either a pro or anti-inflammatory direction. A potential explanation for this duality is maintaining the balance between pro and anti-inflammatory responses and protecting normal tissues; however, more research is needed to elucidate the complex relationship between ascorbate and macrophage differentiation.

#### 2.1.2 Neutrophils

Neutrophils make up to 70% of total leukocytes, and they are the most abundant immune cells in circulation produced at approximately 10^11^ cells per day (44). Neutrophils are the first responders of the innate immune system. They can carry out phagocytosis or degradation of pathogens by ROS and reactive nitrogen species (RNS) production (superoxide, hydrogen peroxide, and nitric oxide) or release their cytotoxic granules. The function of neutrophils has been well-established in innate immune function and antimicrobial activity, and recently, interest in neutrophils as regulators of the tumor microenvironment has developed. Neutrophils have been linked to tumor suppression -via hydrogen peroxide and nitric oxide production- and metastasis promotion (by inducing ROS-mediated DNA damage, IL-1 secretion, and other mechanisms) (45).

Neutrophils maintain high concentrations of ascorbate that can reach up to 14 mM (46). Motility is vital to neutrophils as they must travel to the primary site of infection to eliminate the pathogen or damaged tissue, and exposure to ROS can significantly hinder their motility. *In vitro* and *in vivo* work by Anderson demonstrated that the antioxidant action of ascorbate can rescue neutrophils from ROS exposure and stimulate migration by inhibiting the peroxidase-H_2_O_2_-halide system (47). These high levels of ascorbate promote chemotaxis and stimulate neutrophil superoxide and hydrogen peroxide production, thus allowing them to carry out their phagocytic and killing function once they are at the site of infection (32). *In vitro*, neutrophils treated with 0.1-0.3 mM ascorbate showed a significant increase in ROS production (48). Similarly, human studies with ascorbate supplementation demonstrated improved phagocytic activity and increased ROS production in neutrophils (49, 50).

Neutrophil extracellular traps (NETs) are large web-like extensions of decondensed chromatin with cytosolic and granule proteins that neutrophils form upon stimulation by ROS and the subsequent downstream activation of neutrophil elastase (NE) and protein-arginine deiminase type 4 (PAD4) in a process known as NETosis (51). NETs can also lead to neutrophil death by causing a loss of the nuclear envelope and plasma membrane degradation (51). NETs can promote tumor proliferation, cancer metastasis, and increase the infiltration of immunosuppressive T regulatory lymphocytes into the tumor microenvironment (45, 51, 52). Treating human neutrophils with ascorbate resulted in a decrease in phorbol ester-induced NETosis *in vitro*. In ascorbate deficient mice, NETosis was increased (53). Recently, an *ex vivo* study showed that ascorbate reduced sepsis-induced NETosis in a dose-dependent manner. At 1 mM, it induced a reduction in NETosis, while concentrations > 10 mM increased NETosis (54). These findings suggest that ascorbate is a potential regulator of NETosis which may have beneficial implications for cancer therapy.

#### 2.1.3 Natural killer cells

Natural killer (NK) cells are innate immune granular lymphocytes that kill pathogen infected cells and tumor cells *via* cytotoxic action and produce cytokines such as IFN-γ (55). NK cell depletion is linked to cancer development and a poor prognosis (55). NKs have a unique ability to recognize cancer cells that escape T lymphocytes. 40-90% of human tumors downregulate major histocompatibility complex 1 (MHC-I) molecules used for antigen recognition by CD8^+^ T lymphocytes needed for an immune response (56). However, NK cells can recognize cancer cells regardless of their MHC-I status as they can recognize other surface markers on tumor cells (55, 57), highlighting their importance in anti-tumor immunity and as targets for immunotherapy.

Treating NKs with ascorbate (0.28 mM) increased cell proliferation and promoted the expansion and differentiation of cytokine-stimulated cultures containing NK progenitors without hindering the cytotoxic capacity of the differentiated NKs against leukemia cells under normoxic and hypoxic conditions [46]. In a human toxicology study where NK function was dramatically compromised by toxic chemical exposure (e.g., formaldehyde), 78% of individuals who received ascorbate (0.06 g/Kg body weight dose) showed over a ten-fold enhancement in NK function compared to untreated subjects (58). Similarly, treating functionally-impaired NKs derived from patients with β-thalassemia with 1 mM ascorbate resulted in a significant rescue of NK function compared to untreated controls (59). These data suggest that ascorbate supports NK survival and function. The susceptibility of NKs to oxidative stress (60) – a classic feature of the tumor microenvironment- suggests that ascorbate functions as an antioxidant that can protect NKs in the tumor microenvironment and enable them to carry out their anti-tumor function.

### 2.2 Ascorbate and the adaptive immune system

#### 2.2.1 B lymphocytes

B lymphocytes carry out the humoral immune response. They differentiate into antibody-secreting plasma cells and memory B-cells that play a role in a prolonged immune response. Ascorbate accumulation has been described in B lymphocytes suggesting its functional relevance in humoral immunity (61). Anti-µ-primed spleen-derived B lymphocytes showed enhanced differentiation into plasma cells represented by increased IgM production and increased viability upon treatment with ≥ 0.5 mM 2-O-α-D-glucopyranosyl-L-ascorbic acid (a stable derivative of ascorbate) compared to untreated controls (62). A more recent study demonstrated that treating B-lymphocytes with 0.002-0.01 mM ascorbate promotes plasma cell differentiation by enhancing TET2/3-Mediated DNA demethylation; a similar response was reproduced in animals receiving 4 mg/g body weight ascorbate (18). In a study by Woo et. al, activated B-cells treated with 0.0625 mM – 1 mM ascorbate demonstrated no changes in proliferation but apoptosis was induced in a dose-dependent manner *in vitro* (63).

In human studies, evidence of ascorbate’s role in B cell function is conflicting. Vallance, Prinz, and colleagues demonstrated that vitamin C supplementation (1 g daily) increased IgG and IgM plasma levels suggesting an association between ascorbate and B cell differentiation and function (64, 65). In contrast, Kennes and colleagues found that ascorbate (0.5 g daily) did not affect immunoglobulin levels (66). However, Kennes used a lower dose of ascorbate compared to Prinz and tested an older patient population, which could justify the contradictory findings between the two studies. Hence, more investigation is needed to elucidate the complex role of ascorbate in mediating humoral immunity.

#### 2.2.2 T lymphocytes

T-lymphocytes are responsible for cellular immune response through the action of CD4^+^ T helper cells (Th) and CD8^+^ cytotoxic T cells (Tc) (67). CD4^+^ T cells differentiate into different subsets such as Th1, Th2, Th17, and T Regulatory cells (Treg) (67). Th1 cells drive a pro-inflammatory cell mediated immune response by secreting IFN-γ, IL-2, TNF, and Th2 cells carry out an inflammatory immune response mediated by the cytokines IL4, IL-5, IL-6, IL-10, and IL-13 (67). Th17 cells are pro-inflammatory cells that produce IL-17 and IL-22 and some studies indicate that they may play a role in tumorigenesis (67, 68). Treg cells regulate immune cells by a number of mechanisms including secreting suppressive cytokines such as IL-10 and TGF- β, inducing apoptosis, or using cytokine deprivation to directly suppress other CD4^+^ and CD8^+^ T cells to downregulate cell-mediated immune response (67, 69). Tc cells secrete pro-inflammatory cytokines TNF and IFN-γ that have cytotoxic effects on tumor cells, further, Tc cells can directly kill cells through secretion of perforin and granzyme B that generate pores in the plasma membrane and activate cellular caspases, respectively (67, 70). All T cell subsets and their respective cytokines play essential roles in tumor immunity (67, 70), and major cancer-killing role of T lymphocytes has made them the target of the majority of immunotherapies such as CAR-T (chimeric antigen receptor) cell therapy and ICIs that target PD-1 and CTLA-4 on T cells (71).

Like macrophages, neutrophils, and B cells, T cells accumulate high millimolar levels of ascorbate (72, 73). Ascorbate is vital for numerous cellular functions in the life of a T cell, from development and maturation, proliferation and differentiation, to activation and regulation. When it comes to thymic T cell maturation and development, Manning and colleagues treated T cells with 0.03 mM ascorbate and observed increased T cell maturation, enhanced T cell receptor selection, and CD8 coreceptor upregulation. Similarly, ascorbate-deficient mice showed impaired T cell maturation compared to mice that accumulate ascorbate (73). The Huijskens group treated T cell progenitors with 0.095 mM ascorbate and found it induced T cell maturation and promoted differentiation of double-positive (CD4^+^CD8^+^) thymocytes in the absence of stromal cells, and that ascorbate is required for T cell development when stromal cells are present (74).

As for peripheral T cells, *in vitro*, ascorbate acted as an inhibitor of multiple forms of T cell apoptosis, such as growth factor withdrawal, spontaneous apoptosis, and steroid-induced death (75). Some evidence suggests a potential dose-dependent effect of ascorbate on T cell viability and proliferation. Low ascorbate concentrations (0–0.125 mM) had no effect on T cell viability and proliferation while higher concentrations (0.25-0.5 mM) decreased both (76, 77). Ascorbate polarizes Th cells toward the Th1 phenotype (78, 79). Similarly, naïve T cells had a preference towards differentiating into Th1 cells in the presence of IL-12 produced by dendritic cells treated with 0.08 – 2 mM ascorbate (80). Moreover, dendritic cells treated with 0 – 2 mM ascorbate had elevated IL-12 production that to led increased effector CD8^+^ differentiation and activation causing improved tumor killing *in vivo* (81). With regard to Treg cells, studies show that ascorbate may promote the conversion of effector γδ T cells into Treg cells t through the TET-mediated epigenetic regulation of Foxp3 expression through DNA demethylation, suggesting increased immune-suppressive functions (82, 83). In contrast, Oyarce and colleagues reported that although ascorbate promotes the differentiation of Tregs, their ability to suppress immune response against skin allograft was diminished (84). The impaired Treg function observed by Oyarce is consistent with suppressed Treg function and increased effector T cell activation with ascorbate supplementation in ascorbate-deficient mice as reported by Maeng and colleagues (85).

The first evidence on pharmacological ascorbate as a modulator of anti-tumor immunity in humans was reported in a clinical trial where NSCLC patients received pharmacological ascorbate with carboplatin and paclitaxel, and those with progression-free survival ≥ 6 months showed enhanced CD8^+^ activation by 4.2 fold compared to 1.6 fold in patients with progression-free survival < 6 months (86). Through its role in regulating T cell maturation, differentiation, and activation, ascorbate is not only an immune modulator, but also a very promising regulator T cell mediated antitumor immunity. More investigation is needed in this area in order to elucidate the multifaceted function of ascorbate in immune response and clearly define the role of dose and timing on immune cell proliferation and activation.

## 3 Ascorbate and cancer therapy

Cameron and Pauling lead clinical trials on ascorbate and cancer in the 1970s. Terminal patients were treated with high dose intravenous and oral ascorbate and it was found that ascorbate supplementation prolonged survival by 210-300 days and 22% of ascorbate-treated patients had greatly increased survival time (over 2.4 years) compared to matched controls (87, 88). Subsequent clinical trials with oral ascorbate in patients with advanced colorectal, lung, stomach, liver, and pancreatic cancer failed to reproduce Cameron and Pauling’s findings (89, 90). Unfortunately, this decreased the interest in ascorbate as an anti-cancer therapy. A pivotal criticism of these trials is that they did not use intravenous ascorbate which was later shown to have a pharmacokinetic ability to increase plasma ascorbate concentrations to the tens of milli-molar range (91). Multiple studies by Levine and colleagues then confirmed that for cancer cell killing to be accomplished, pharmacological doses of ascorbate are required as they lead to the generation of toxic concentrations of hydrogen peroxide in the extracellular space in the tumor but not in the blood leading to selective killing (92–94). These findings initiated a new wave of ascorbate research in cancer therapy in the early 2000s (7, 95–100).

A growing body of evidence shows that ascorbate can selectively kill cancer cells by acting as a prooxidant by generating high fluxes of hydrogen peroxide that can react with intracellular labile iron *via* Fenton chemistry while it is believed to simultaneously protect normal tissue by acting as a donor antioxidant (26, 93). Chen and colleagues showed that ten cancer cell lines had an EC50 of < 4 mM with ascorbate while the non-malignant cell lines could survive ascorbate concentrations up to 20 mM (93).

These findings were confirmed *in vivo* in xenograft models of ovarian, pancreatic, and glioblastoma (GBM) cancers where pharmacological ascorbate significantly reduced tumor growth (93). These findings inspired the investigation of the potential of ascorbate as a sensitizing agent in combination with chemotherapy and radiation. In pancreatic cancer, pharmacological ascorbate was a radiosensitizer *in vitro* and in a xenograft mouse model (95). Concordantly, clinical trial data suggests that pharmacological ascorbate increases overall survival in patients with pancreatic cancer receiving concurrent chemotherapy (97). As a chemosensitizer, pharmacological ascorbate exhibited a synergistic effect when combined with gemcitabine *in vitro* and pre-clinical pancreatic cancer models (101). Consistent with these results, subsequent early phase clinical trials showed that pharmacological ascorbate is safe with promising clinical outcomes when combined with gemcitabine (102, 103). Additional early phase clinical trials in GBM, non-small cell lung carcinoma (NSCLC), and ovarian cancer also found pharmacological ascorbate to be safe with promising clinical outcomes (96, 98, 104, 105). In addition to enhancing radiation and chemotherapy effectiveness, pharmacological ascorbate has also significantly reduced chemotherapy induced normal tissue toxicity as reported by Ma and colleagues in ovarian cancer, and radiation-induced normal tissue injury as reported by Alexander and colleagues in pancreatic cancer (97, 104). Taken together, data from all the aforementioned *in vitro*, pre-clinical, and clinical studies demonstrate its excellent capacity to kill cancer cells, improve response to conventional therapy -even in cancers known for poor response and poor prognosis such as pancreatic cancer-, and minimize the normal tissue damage associated with chemotherapy and radiation. With our current understanding of the importance of tumor immunology, ascorbate may increase the efficacy of ICIs.

## 4 Ascorbate as a sensitizer for cancer immunotherapy

While multiple basic and clinical studies focused on ascorbate and its clinical application in the context of chemotherapy and radiotherapy, very few studies have explored its potential as a sensitizer for cancer immunotherapy. The roles that ascorbate plays in immune regulation previously discussed in this review, along with its prooxidant action against cancer and antioxidant protective action in normal tissues, suggest pharmacological ascorbate may enhance the response of cancer cells to ICIs and protect against ICI-induced normal tissue injury such as pneumonitis.

Luchtel and colleagues evaluated pharmacological ascorbate (1.5 M) in a mouse lymphoma model in combination with anti-PD-1. This study showed an increase in lymphoma immunogenicity represented by a 15%- 21% increase in Tc-mediated cell killing (106). Another finding from this study was that combining high doses of ascorbate with ani PD-1 significantly increased Tc and macrophage infiltration in the tumor microenvironment and increased IL-12 production, increased Tc and NK activation, and increased granzyme B production (106). Lastly, this group showed that combining pharmacological ascorbate (4g/kg) with anti-PD-1 has a synergistic effect that significantly decreased tumor growth compared to untreated controls or treatment with single agents (106). Magrì, A., et al. evaluated mouse models of melanoma, breast, pancreatic, and colorectal cancers to test the effect of ascorbate in combination with anti-PD-1 and anti-CTLA-4. First, they examined the effect of ascorbate on anti-tumor immune response and found it is primarily a Tc mediated process emphasizing the potential of ascorbate sensitizing against anti-PD-1 and anti-CTLA-4 (107). Consistent with Luchtel’s group, they reported significantly increased Tc infiltration in the tumor microenvironment and reduced tumor growth when combining ascorbate with anti-PD-1 and anti-CTLA-4 and (107). Tumor volume was significantly reduced in breast, colorectal, and pancreatic cancers by the ascorbate anti-PD-1 and anti-CTLA-4 combinations (107). Mice that showed complete response exhibited progression-free survival for up to a year (107). Most recently, a study reported a synergistic effect in a mouse model of renal cell carcinoma treated with high dose (0.5 g/kg) ascorbate combined with anti PD-L1 (17). Compared to untreated controls, high dose ascorbate alone, or anti PD-L1 alone, the combination showed the highest levels of CD4^+^ and CD8^+^ infiltration in the tumors, in addition to elevated IFN-γ and IFN-γ-induced CXCL9, CXCL10 and CXCL11 (17). Interestingly, the expression levels of PD-L1 were significantly increased by high dose ascorbate, suggesting that it can potentially make tumors more responsive to checkpoint blockade (17). These initial findings describing pharmacological ascorbate and cancer immunotherapy, ICIs in particular, are promising and warrant further investigation.

## 5 Discussion and future directions

**Figure 1:**
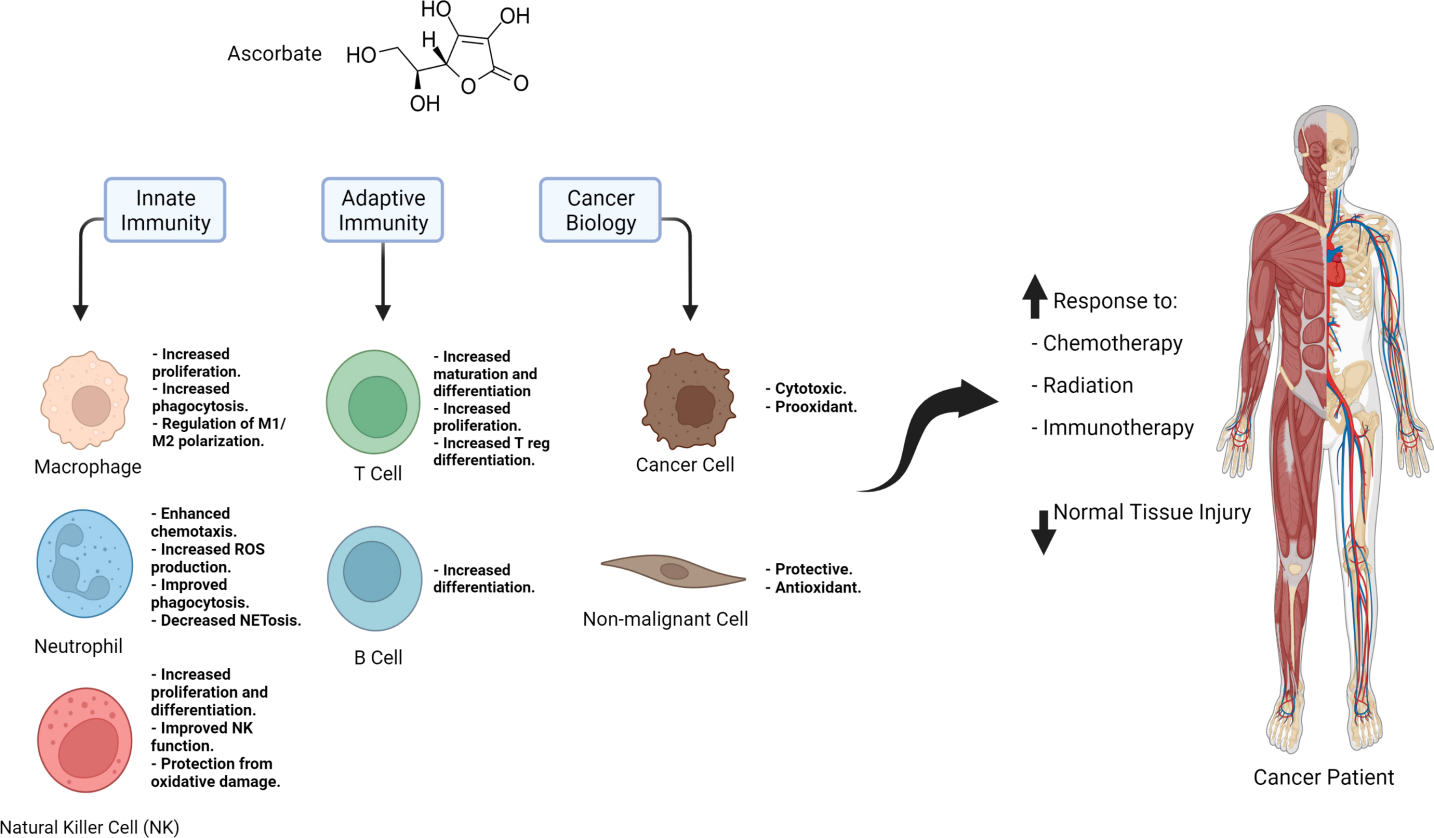
Summary of the immune regulatory effects of ascorbate, the role it plays it cancer and non-malignant cells, and its potential applications in cancer therapy.

Through its antioxidant activity and epigenetic regulation, ascorbate is a crucial modulator of immune function and the anti-tumor immune response **(** **Figure 1** **)**. Moreover, as a small molecule antioxidant, it protects normal tissue against ROS. Metabolic changes in cancer cells, such as their reduced ability to metabolize hydrogen peroxide and their increased iron accumulation, make them susceptible to the prooxidant action of ascorbate. As a result, pharmacological ascorbate has been recently exploited in multiple clinical trials as a chemo- and radiosensitizer, but the field of ascorbate and cancer immunotherapy has yet to be explored significantly. Three studies showed pharmacological ascorbate combinations with ICIs: a) increase Tc infiltration in the tumor microenvironment and b) exert synergistic effects on tumor suppression. These findings emphasize the importance of this novel approach to cancer therapy. Future studies should include other malignancies and clinically relevant combinations such as using ICIs with chemotherapy and radiation. Additionally, future studies should address how pharmacological ascorbate affects primary and acquired resistance to ICIs, and whether or not it protects normal tissue from adverse effects associated with ICIs such as pneumonitis and colitis. Mechanistic studies that elucidate the route of action of ascorbate as a sensitizer to ICIs are also critical. A noteworthy potential mechanism that can be studied in this context is the epigenetic regulation of TET2; prior research suggests that ascorbate promotes TET2 activation and leads to the expression of genes associated with anti-tumor immunity (19). Peng and colleagues explored this mechanism in their study and showed that with the loss of TET, high dose ascorbate lost the synergistic effect it had with anti PD-L1 in a mouse model of renal cell carcinoma, thus emphasizing the importance of epigenetic regulation as a focus for mechanistic studies on ascorbate and immunotherapy (17). There are many questions that need to be answered about ascorbate and cancer immunotherapy. Answering these questions will help expedite the establishment of clinical trials that will create a new and potentially improved standard of care regimens for cancer patients.

## Author contributions

AZ and BA wrote the manuscript. LS, AM, SH, JS, MP, ZZ, TW, VM, JC, MF, JH, SV, and DS provided expertise and feedback. BA, DS, MF, SV, and JH provided funding. All authors contributed to the article and approved the submitted version.

## Funding

This work was supported by NCI P30CA086862, Gateway for Cancer Research award G-17-1500T32 GM007337, UIHC-CCOM Iowa Aging Initiative, NIAID R21AI157121, NIH/NCI T32CA078586, NCI P01CA217797, and the Holden Comprehensive Cancer Center Gift Funds.

## Conflict of interest

The authors declare that the research was conducted in the absence of any commercial or financial relationships that could be construed as a potential conflict of interest.

## Publisher’s note

All claims expressed in this article are solely those of the authors and do not necessarily represent those of their affiliated organizations, or those of the publisher, the editors and the reviewers. Any product that may be evaluated in this article, or claim that may be made by its manufacturer, is not guaranteed or endorsed by the publisher.
